# Genetic Regulation of Adult Stature in Humans

**DOI:** 10.1210/clinem/dgaa210

**Published:** 2020-04-17

**Authors:** Allen W Root

**Affiliations:** Adjunct Professor of Pediatrics, Johns Hopkins School of Medicine; Professor of Pediatrics Emeritus, University of South Florida Morsani College of Medicine; Division of Pediatric Endocrinology and Diabetes, Johns Hopkins All Children’s Hospital, St. Petersburg, Florida

**Keywords:** fibrillin 1, genetic regulation of stature, growth hormone, height

In this issue of the Journal of Clinical Endocrinology & Metabolism, Lin et al (1) examine the genetic profile of Taiwanese Han Chinese adult women and men with familial (genetic) short stature (FSS), defined as fully grown height below the third percentile for this population. Utilizing genome-wide association studies (GWAS), they identify in this population 10 previously unreported single nucleotide polymorphisms (SNPs) related to 5 genes or pseudogenes; the other 5 sites had no defined specifically associated genes. The authors utilized these 10 SNPs to calculate a “polygenic risk predisposition score” for FSS. Additionally, the authors found 9 previously identified SNPs/genes associated with FSS in this population. The 5 genes that were uniquely related to short stature in the Han Chinese encoded: an uncharacterized noncoding RNA (*LOC105374144*); *COL6A5*—one of 6 members of the collagen 6 family present in the pericellular matrix of cartilage; *UGT2B17*—encoding an uridine diphosphate glucuronosyltransferase that catalyzes the glucuronidation of many steroid hormones thereby increasing their solubility and their rate of urinary excretion; *IQCM*—a gene of uncertain function; *PGM5P2*—encoding a “pseudogene,” possibly a nonfunctional segment of DNA. Of the 9 previously recognized genes associated with FSS and also identified in the Han Chinese, none were involved directly with chondrocyte differentiation or activity or cartilage formation, but they were associated with basic cellular processes such as cell division (*ANAPC13, ATF7, CDK7, CABLES1*), epigenetic gene-regulatory activity (*LCORL*), membrane function (*DNM3*), intracellular signaling (*GPR126*), sulfhydryl oxidation (*QOSX2*), and mitochondrial function (*UQCC1*).

An individual’s adult height reflects the impact of multiple factors—foremost of these is her/his genetic composition, which under optimal circumstances accounts for approximately 75% to 80% of the variability in normal adult stature; in addition, socioeconomic status, nutrition, illness/injuries/emotional insults/other adverse events, hormonal milieu, and evolutionary factors also influence attainment of genetically endowed stature (2-4). More than 3200 genetic variants at greater than 700 genomic loci (usually in noncoding DNA intergenic or intronic sequences) influence linear growth and adult stature in subjects of European ancestry (5). The intergenic nucleotide variants are often the sites of epigenetic regulatory control of the expression and rate of transcription of the associated genes. Each allelic variant usually accounts for a very small fraction (± 0.2 cm) of adult stature, although variants of a few genes (*AR, CHSY1, CRISPLD2, IHH, STC2*) may affect adult height by as much as 2 cm (4, 6). Approximately half of the allelic variants are associated with increased adult stature and half with decreased mature height. The products of the identified height-influencing genes regulate embryogenesis, differentiation, multiplication, maturation, senescence, and apoptosis of growth plate chondrocytes, ossification of the cartilaginous growth plates at either end of most long bones and those of the spinal vertebrae, and the metabolic processes that enable and control them (although often at points in the regulatory pathways far distant from the targeted chondrocyte itself), as well as the regulatory effects of osteoclasts upon cartilage mineralization.

There are a number of genes in the growth hormone axis that exert profound adverse effects upon linear growth, while variants of some of these genes also affect normal adult stature (3, 4, 7-9). Thus, many members of the family of genes that direct embryogenesis of the adenohypophysis (*POU1F1, PROP1, LHX4, HESX1*) or regulate the synthesis and functional activity of growth hormone (GH) (i.e., *GHRHR, GH1, GHSR, GHR, STAT5B, IGF1, IGF2, IGF1R*) also influence normal adult height. Variants of *THRB*, encoding the beta subunit of the thyroid hormone receptor beta, *AR* encoding the androgen receptor, and *PTHR1*, encoding the receptor for parathyroid hormone/parathyroid hormone-related protein, also influence adult stature (6). Gene variants that affect stature in normal subjects, as well as impede growth in patients with short stature when genetically mutated, include those that impair intracellular signaling (*GNAS* [Albright hereditary osteodystrophy], *RAF1* [LEOPARD and Noonan syndromes], and *WNT5A* [Rabinow syndrome]); transcription (*SOX9* [campomelic dysplasia] and *CREBBP* [Rubenstein-Taybi syndrome]); DNA repair (*FANCA* [Fanconi anemia] and *DNA2* [Seckel syndrome]); chromatin remodeling (*ARD1B* [Coffin-Siris syndrome]); sister chromatid cohesion (*NIPBL* [Cornelia de Lange syndrome]); collagen formation (*FBN1* [acromicric dysplasia], *COL11A1* [fibrochondrogenesis], *COL9A2* [multiple epiphyseal dysplasia], and *ACAN* [spondyloepimetaphyseal dysplasia]); and paracrine signaling (*NPR2* [acromesomelic dysplasia] and *GDF5* [brachydactyly, types A1, A2]) (2).

Deleterious mutations in *FBN1*, encoding fibrillin 1—a cartilage matrix protein—may be associated with either autosomal dominant tall stature-arachnodactyly-lax joints (Marfan syndrome) or extremely short stature-brachydactyly-stiff joints (acromicric dysplasia, geleophysic dysplasia 2, Weill-Marchesani syndrome 2). Marfan syndrome is the consequence of mutations throughout *FBN1* that decrease the synthesis and quantity of fibrillin 1, while acromicric dysplasia and related disorders are the result of variants of *FBN1* restricted to exons 41 and 42 (e.g., Tyr1700Cys, Ser1750Arg) the gene site that encodes the transforming growth factor (TGF)β-binding-protein-like domain 5 of fibrillin 1. In patients with either Marfan syndrome or acromicric dysplasia there is reportedly an increase in bioactive TGFβ due to its decreased binding by fibrillin 1, which is responsible in part for the clinically disparate manifestations of these disorders, although the mechanism(s) that underlie the opposite clinical manifestations of these disorders remains unknown at present (10). Perhaps an abnormality in the complex processing of TGFβ after its secretion may account for the disparate biological effects of TGFβ in these distinctly clinically opposite disorders (11).

*STC2* encodes stanniocalcin 2, a protein present in bone, which impairs the biologic activity of pregnancy-associated plasma protein A (encoded by *PAPPA*). PAPP-A is a proteinase, one of whose functions is to degrade insulin-like growth factor binding proteins (IGFBP)-4 and IGFBP-5, thereby releasing insulin-like growth factor 1 (IGF-1) and increasing its free levels thus enabling its binding to the IGF-1 receptor (IGF1R). An SNP preceding *STC2* is associated with an increase in adult height of approximately 2.1 cm, suggesting this variation leads to decreased expression of *STC2* resulting in increased expression of *PAPPA*, enhanced proteolysis of IGFBP-4 and IGFBP-5, and increased levels of free IGF-I available to bind to IGF1R, thereby increasing chondrocyte proliferation (12, 13).

In addition to the mutations in *FBN1* associated with Marfan syndrome, pathologically tall stature may be related to variants of *TGFBR1/TGFBR2* (Loeys-Dietz syndrome, a disorder with Marfanoid features in which excessive activity of TGFβ is also of pathophysiologic relevance), *NSD1* (cerebral gigantism/Sotos syndrome), *EZH2* (Weaver syndrome), *DNMT3A* (Tatton-Brown-Rahman syndrome), and *NFIX* (Malan syndrome) (14). Tall stature may also be associated with the triple X (47XXX), fragile X, and Klinefelter (47XXY) syndromes, homocystinuria due to mutations in CBS encoding cystathionine beta-synthase, and inactivating variants of *ER* encoding the estrogen receptor.

Unraveling of the basic biologic roles of the plethora of genes that influence adult height and of their variants that adversely affect this process has provided unprecedented insight into the fundamental aspects of the complex phenomenon of linear growth. Major future challenges include: (1) the functional integration of the pathways and the mechanisms and timing of the interaction of these myriad gene products upon the growth process, and (2) the translation of this information into therapies that effectively treat those patients with gene variants that perversely influence this system.

## Abbreviations

FSS: familial short stature
GWAS: genome-wide association study
IGF-1: insulin-like growth factor 1
IGFBP: insulin-like growth factor binding protein
IGF1R: insulin-like growth factor 1 receptor
SNP: single nucleotide polymorphism
TGFβ: transforming growth factor β

## Additional Information

***Disclosure Summary:*** The author has nothing to disclose.
